# Plasminogen activator inhibitor-1 enhances radioresistance and aggressiveness of non-small cell lung cancer cells

**DOI:** 10.18632/oncotarget.8208

**Published:** 2016-03-19

**Authors:** JiHoon Kang, Wanyeon Kim, TaeWoo Kwon, HyeSook Youn, Joong Sun Kim, BuHyun Youn

**Affiliations:** ^1^ Department of Integrated Biological Science, Pusan National University, Busan, 46241, Republic of Korea; ^2^ Department of Biological Sciences, Pusan National University, Busan 46241, Republic of Korea; ^3^ Nuclear Science Research Institute, Pusan National University, Busan 46241, Republic of Korea; ^4^ Department of Integrative Bioscience and Biotechnology, Sejong University, Seoul 05006, Republic of Korea; ^5^ Research Center, Dongnam Institute of Radiological and Medical Sciences, Busan 46033, Republic of Korea

**Keywords:** PAI-1, NSCLC, paracrine, radioresistance, EMT

## Abstract

Acquired resistance of tumor cells during treatment limits the clinical efficacy of radiotherapy. Recent studies to investigate acquired resistance under treatment have focused on intercellular communication because it promotes survival and aggressiveness of tumor cells, causing therapy failure and tumor relapse. Accordingly, a better understanding of the functional communication between subpopulations of cells within a tumor is essential to development of effective cancer treatment strategies. Here, we found that conditioned media (CM) from radioresistant non-small cell lung cancer (NSCLC) cells increased survival of radiosensitive cells. Comparative proteomics analysis revealed plasminogen activator inhibitor-1 (PAI-1) as a key molecule in the secretome that acts as an extracellular signaling trigger to strengthen resistance to radiation. Our results revealed that expression and secretion of PAI-1 in radioresistant cells was increased by radiation-induced transcription factors, including p53, HIF-1α, and Smad3. When CM from radioresistant cells was applied to radiosensitive cells, extracellular PAI-1 activated the AKT and ERK1/2 signaling pathway and inhibited caspase-3 activity. Our study also proposed that PAI-1 activates the signaling pathway in radiosensitive cells via extracellular interaction with its binding partners, not clathrin-mediated endocytosis. Furthermore, secreted PAI-1 increased cell migration capacity and expression of EMT markers *in vitro* and *in vivo*. Taken together, our findings demonstrate that PAI-1 secreted from radioresistant NSCLC cells reduced radiosensitivity of nearby cells in a paracrine manner, indicating that functional inhibition of PAI-1 signaling has therapeutic potential because it prevents sensitive cells from acquiring radioresistance.

## INTRODUCTION

Lung cancer is the most common cancer and the leading cause of cancer-related deaths worldwide [1]. Radiotherapy, alone or in combination with surgery or chemotherapy, plays a major role in management of non-small cell lung cancer (NSCLC). However, resistance to radiation therapy is generally induced by intrinsic mechanisms and extracellular factors through intercellular communication, which greatly reduces the efficacy of radiotherapy for NSCLC. Although previous studies have contributed to a partial understanding of the mechanisms of intrinsic radioresistance, radioresistance acquired via extracellular factors mediating intercellular communication is still not fully understood [2, 3]. Thus, elucidation of the functional relationship among cells within a tumor may reveal novel therapeutic targets for regulation of therapeutic resistance.

Tumors can be initially developed from a single cell possessing genetic alterations. After formation of malignant tumor mass, tumors are composed of cell populations with distinct histological and genetic features that allow each subclone to have differential responsiveness to immunity, metabolism, growth signals, and anti-cancer therapies. This phenomenon is known as intratumoral heterogeneity [4–6]. Intratumoral heterogeneity has recently been elucidated through genomic/proteomic analysis for several cancer types [7, 8]. Multi-focal microdissection analysis with NSCLC patients showed that intratumoral heterogeneity for epidermal growth factor receptor (EGFR) mutation was detected in about 30% of patients, and was significantly associated with disease-free survival after EGFR-specific chemotherapy [7]. Evaluation of 100 primary cancers demonstrated mutational diversity consisting of various cancer-associated genes among individual tumors [8]. In addition, intratumoral communication through direct cell-to-cell contacts and paracrine signaling was shown to play pivotal roles in immune surveillance, apoptosis-resistance, invasion, and angiogenesis in response to various stimuli [9–11]. In small cell lung cancer, paracrine signaling between heterogeneous tumor subclones was required to induce local invasion and intravasation for metastatic conversion [9]. In addition, WNT16B and interleukin-17 provided from tumor stromal cells were identified as paracrine regulators to promote resistance of tumor cells to anti-cancer therapy [10, 11]. Although intratumoral and tumor-stroma interactions that contribute to the neoplasm and tumor malignancy have become a major focus in cancer therapy, the mechanism and key molecules that enhance resistance to therapy between individual tumor cells with different characteristics remains largely unknown.

Plasminogen activator inhibitor-1 (PAI-1) is a well-known factor involved in regulation of intra- and extra-vascular fibrinolysis with urokinase-type plasminogen activator (uPA) and its receptor uPAR. PAI-1 stimulates proteolytic activation of extracellular plasminogen, resulting in the formation of serine protease plasmin, which is able to degrade extracellular matrix (ECM) proteins including fibrin and laminin. This role of PAI-1 in fibrinolysis systems associated with degradation and remodeling of the surrounding tissues is also fundamental to tumor progression, as indicated by expansion of the tumor mass, induction of tumor cell proliferation, invasion, migration, and release of tumor growth factors and cytokines [12]. Increased expression of PAI-1 in tumors has been reported as an informative prognostic marker associated with poor outcome of a number of cancer types, including NSCLC [13, 14]. Elevated levels of PAI-1 and uPA have been reported to be correlated with low levels of disease-free survival in breast cancer subtypes, especially HER2-positive patients [14]. In head and neck squamous cell carcinoma (HNSCC), high PAI-1 levels were found to be closely correlated with perineural invasion and shorter disease-free survival [15]. PAI-1 secreted from mesenchymal stem cells was recently demonstrated to increase migration and proliferation capacity of colon cancer cells [16]. Furthermore, PAI-1 has been reported as a key regulator of tumor aggressiveness and survival that mediates several intracellular signaling pathways. The association of PAI-1 with uPA/uPAR complex can induce activation of PI3K/AKT, focal adhesion kinase (FAK), and extracellular signal-regulated kinase (ERK) signalings leading to cell survival and proliferation [17–19]. In addition to the recognized function of PAI-1 in tumor progression, some reports have suggested that PAI-1 could facilitate epithelial-mesenchymal transition (EMT) through the regulation of EMT-associated proteins, such as E-cadherin, Snail, and Vimentin [20]. These data provide crucial evidence demonstrating that PAI-1 as a secreted protein may significantly enhance tumor malignancy, including tumor metastasis and resistance to therapy in a paracrine manner.

To elucidate the mechanism and key molecules in intercellular communication that induce resistance to radiotherapy, we screened changes in the secretome in IR-irradiated radioresistant NSCLC cells. Radiation-dependent secretion of PAI-1 from radioresistant NSCLC cells was shown to activate survival signaling and EMT induction in radiosensitive NSCLC cells. Our findings provide a possible explanation of how radiosensitive cells can acquire resistance to radiation and promote the EMT phenotype through interaction with neighboring radioresistant cells in a paracrine manner. Furthermore, we suggest that PAI-1 could be a promising therapeutic target for enhancing the efficiency of radiotherapy to treat lung cancer.

## RESULTS

### Secretome of radioresistant cells promotes survival of radiosensitive cells in NSCLC

**Figure 1:**
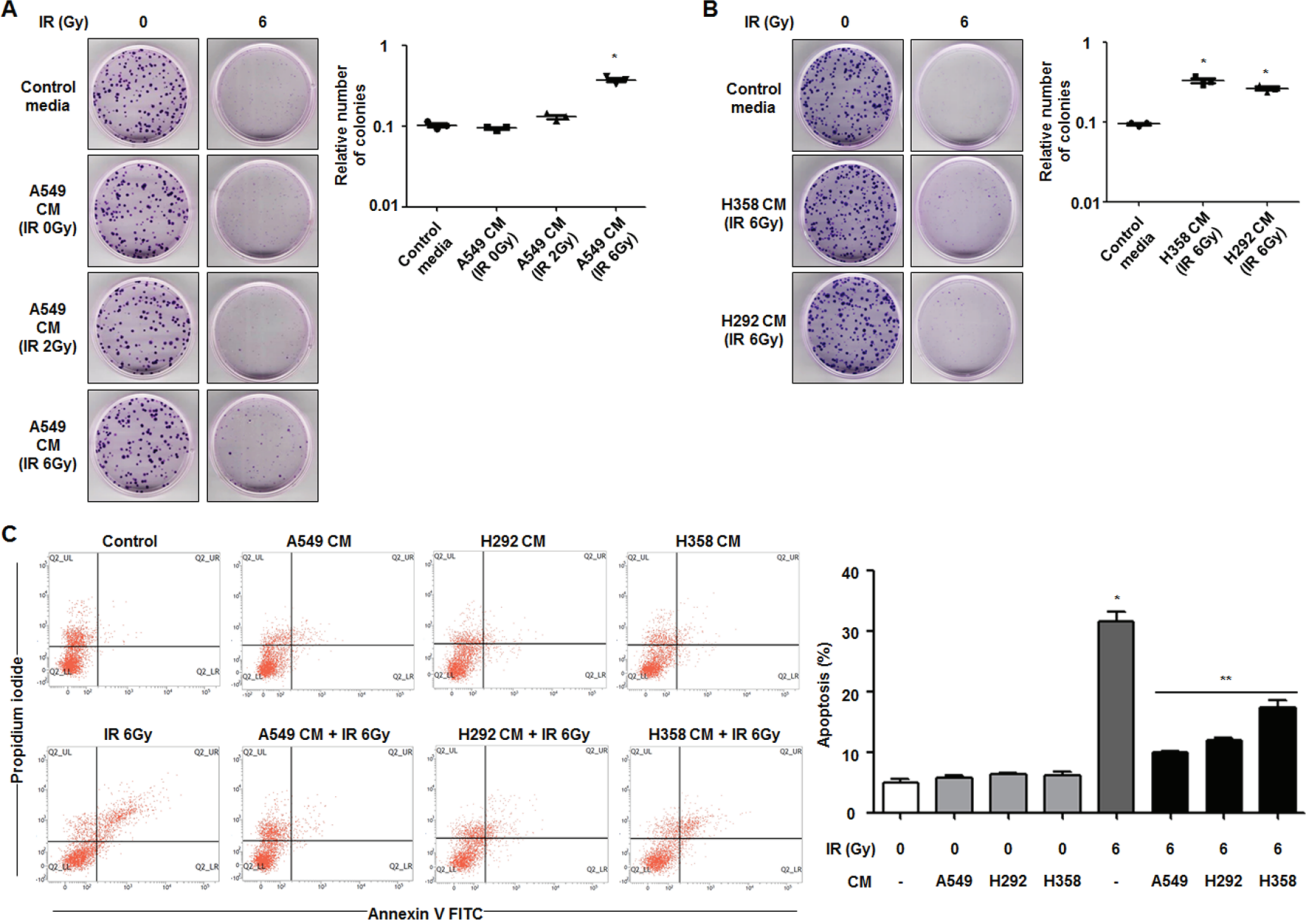
Secretome from radioresistant cells promotes survival of radiosensitive cells in NSCLC (**A**) Effects of CM derived from radioresistant A549 cells on survival of radiosensitive NCI-H460 cells in response to radiation were measured by a colony forming assay. The relative numbers of colonies of NCI-H460 cells are indicated in the graph. *p < 0.05 compared with irradiated cells treated with control media. (**B**) Effects of CM derived from other radioresistant cells, NCI-H358 and NCI-H292 cells, on survival of NCI-H460 cells in response to radiation were measured by a colony forming assay. *p < 0.05 compared with irradiated cells treated with control media. (**C**) Effects of CM derived from A549, NCI-H358, or NCI-H292 cells on radiation-induced apoptosis of NCI-H460 cells were analyzed by an Annexin V staining assay. The relative levels of Annexin V- and PI-positive populations of NCI-H460 cells are indicated in the graph. *p < 0.05 compared with non-irradiated cells; **p < 0.05 compared with irradiated cells treated with control media.

To investigate whether cells with radiosensitive characteristics can become radioresistant via intercellular communication, A549 and NCI-H460 cells were selected as relatively radioresistant cells and radiosensitive cells, respectively [21]. After preparation of CM from radioresistant A549 cells, we first measured the proliferation capacity of radiosensitive NCI-H460 cells by a colony forming assay after irradiation with or without treatment of CM. Colony formation of NCI-H460 cells increased by ~3.70 fold in response to treatment with CM obtained from irradiated A549 cells compared to the group treated with control media in response to 6 Gy of irradiation (Figure 1A). As shown in Figure 1B, the populations of NCI-H460 cells under radiation exposure also increased in response to treatment with CM harvested from irradiated NCI-H358 or NCI-H292 cells, which were shown to be radioresistant cell lines in a previous study [22]. However, CM obtained from relatively radiosensitive cell lines, including NCI-H460, NCI-H157, NCI-H23 cells, had no effects on cell survival after irradiation (Supplementary Figure S1A).

To determine whether the secretome of CM from radioresistant cells influences radiation-induced apoptosis in radiosensitive cells, NCI-H460 cells incubated with CM from irradiated A549, NCI-H292, or NCI-H358 cells for 6 h were irradiated and subsequently analyzed by an Annexin V-FITC/PI staining assay. When NCI-H460 cells were treated with fresh serum-free control media, the population of Annexin V-positive and PI-positive cells after 6 Gy of irradiation was about 30% of the total population. However, NCI-H460 cells treated with CM from A549, NCI-H292, or NCI-H358 cells showed significantly decreased cell death in response to 6 Gy of irradiation (Figure 1C). On the other hands, NCI-H460 cells treated with CM from NCI-H460, NCI-H157, NCI-H23 cells showed similar cell death rate in response to irradiation compared to the groups treated with control media (Supplementary Figure S1B). Taken together, these data suggest that CM from radioresistant cells can increase cell proliferation and decrease cell death in irradiated NCI-H460 cells, a relatively radiosensitive cell line.

### PAI-1 in secretome of IR-irradiated radioresistant cells increases survival of IR-irradiated radiosensitive cells in NSCLC

**Figure 2:**
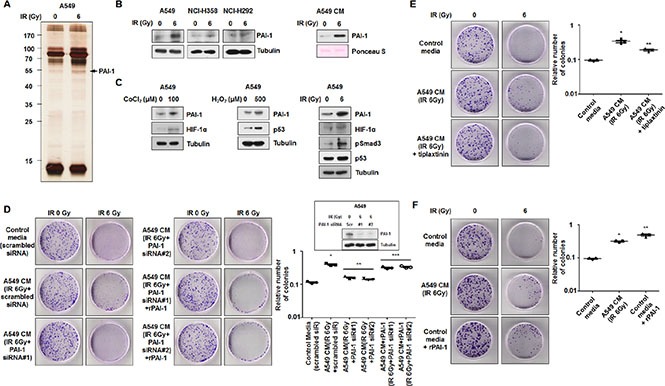
PAI-1 secreted from radioresistant cells under irradiation is a key paracrine factor in survival of radiosensitive cells in NSCLC (**A**) The secretomes in CM of A549 cells exposed to 6 Gy were analyzed by silver staining and mass spectrometry. The band indicated by an arrowhead corresponds to PAI-1, which was increased by irradiation. (**B**) Radiation-induced expression levels of PAI-1 in A549, NCI-H358, and NCI-H292 cells and CM of A549 cells were analyzed by Western blotting. (**C**) Expression levels of PAI-1 and several transcription factors in A549 cells under hypoxia, ROS, or IR were analyzed by Western blotting. (**D**) The effects of PAI-1 knockdown on survival of NCI-H460 cells in response to radiation were measured by a colony forming assay using PAI-1-specific siRNA. The inset shows that siRNA oligonucleotides specific for PAI-1 significantly reduced PAI-1 expression in A549 cells measured by Western blotting. *p < 0.05 compared with irradiated cells treated with control media; **p < 0.05 compared with irradiated cells treated with CM of A549 cells without treatment of PAI-1 siRNA. (**E**) Effects of PAI-1 activation on survival of NCI-H460 cells in response to radiation were measured by a colony forming assay using tiplaxtinin (20 μM), a PAI-1 specific inhibitor. *p < 0.05 compared with irradiated cells treated with control media; **p < 0.05 compared with irradiated cells treated with CM of A549 cells not containing tiplaxtinin. (**F**) The effects of PAI-1 levels on survival of NCI-H460 cells in response to radiation were confirmed by a colony forming assay using rPAI-1 (50 ng/ml). *p < 0.05 compared with irradiated cells treated with control media; **p < 0.05 compared with irradiated cells treated with CM of A549 cells.

To identify the key factors that made NCI-H460 cells more resistant to radiation, we analyzed the secretome from irradiated A549 cells. CM from non-irradiated or irradiated A549 cells were assessed by a silver-staining assay, and the identities of several proteins were determined by peptide mass fingerprint with high confidence (Figure 2A, Supplementary Figure S2). PAI-1 was identified as a candidate for a paracrine factor that mediates intercellular communication between A549 and NCI-H460 cells. Datasets available from Oncomine (http://www.oncomine.org) and cBioportal (http://www.cbioportal.org) presented that the expression of *SERPINE1* in lung tumors was not significantly elevated compared to normal lung (Supplementary Figure S3A) and that gene amplification (1.72 ± 0.58%), mutation (1.8 ± 0.46%), or deletion (0.07 ± 0.07%) of *SERPINE1* were detected in NSCLCs (Supplementary Figure S3B), respectively [23–25]. It indicated that genetic alterations of *SERPINE1* were present, but rare in NSCLCs. Thus, we hypothesized that PAI-1 expression might be induced in response to extracellular stimuli such as radiation, leading to tumor radioresistance and progression. To confirm the involvement of PAI-1 in radiation, we measured the expression of PAI-1 in response to radiation in NSCLC cell lines. Expression of PAI-1 increased in irradiated A549, NCI-H358, and NCI-H292 cells, and PAI-1 was subsequently released from A549 cells into the media (Figure 2B). However, expression of PAI-1 did not increase in irradiated NCI-H460, NCI-H157, and NCI-H23 cells, and secreted PAI-1 was not detected in the media obtained from NCI-H460 cells (Supplementary Figure S4). The expression of PAI-1 has been shown to be elevated by several transcription factors, including HIF-1α, p53, and phospho-Smad3, which were activated in response to stress conditions such as hypoxia and oxidative stress, as well as radiation exposure [26, 27]. To determine whether the expression of PAI-1 was increased by hypoxia or reactive oxygen species (ROS), we measured the protein levels of PAI-1 and associated transcription factors in A549 cells after treatment with radiation, CoCl_2_, or H_2_O_2_. We found that PAI-1 was induced under hypoxia or high ROS levels (Figure 2C). In addition, the protein levels of HIF-1α, p53, and phospho-Smad3 in A549 cells also increased in response to radiation exposure. To determine whether PAI-1 released from A549 cells is a key factor that made NCI-H460 cells more radioresistant, CM obtained from A549 cells treated with two PAI-1-specific siRNAs prior to irradiation was applied to NCI-H460 cells. The increase in NCI-H460 cells was blocked, resulting in levels similar to that of cells treated with control media under radiation exposure (Figure 2D). These results were recovered by treatment of recombinant PAI-1 (rPAI-1). In addition, treatment of NCI-H460 cells with tiplaxtinin, a PAI-1 inhibitor, in conjunction with CM of A549 cells resulted in reduced numbers of NCI-H460 cells in response to irradiation (Figure 2E). To confirm the role of PAI-1 on colony formation of H460 cells, rPAI-1 was administered to NCI-H460 cells. Similar to the group treated with CM of A549 cells, colony formation of NCI-H460 cells was significantly increased by rPAI-1 treatment (Figure 2F). These results indicated that radioresistance of radiosensitive cells was acquired by radiation-induced extracellular PAI-1 from nearby radioresistant cells.

### Secreted extracellular PAI-1 increases radioresistance of NCI-H460 cells through activation of AKT and ERK1/2 and inhibition of caspase-3

**Figure 3:**
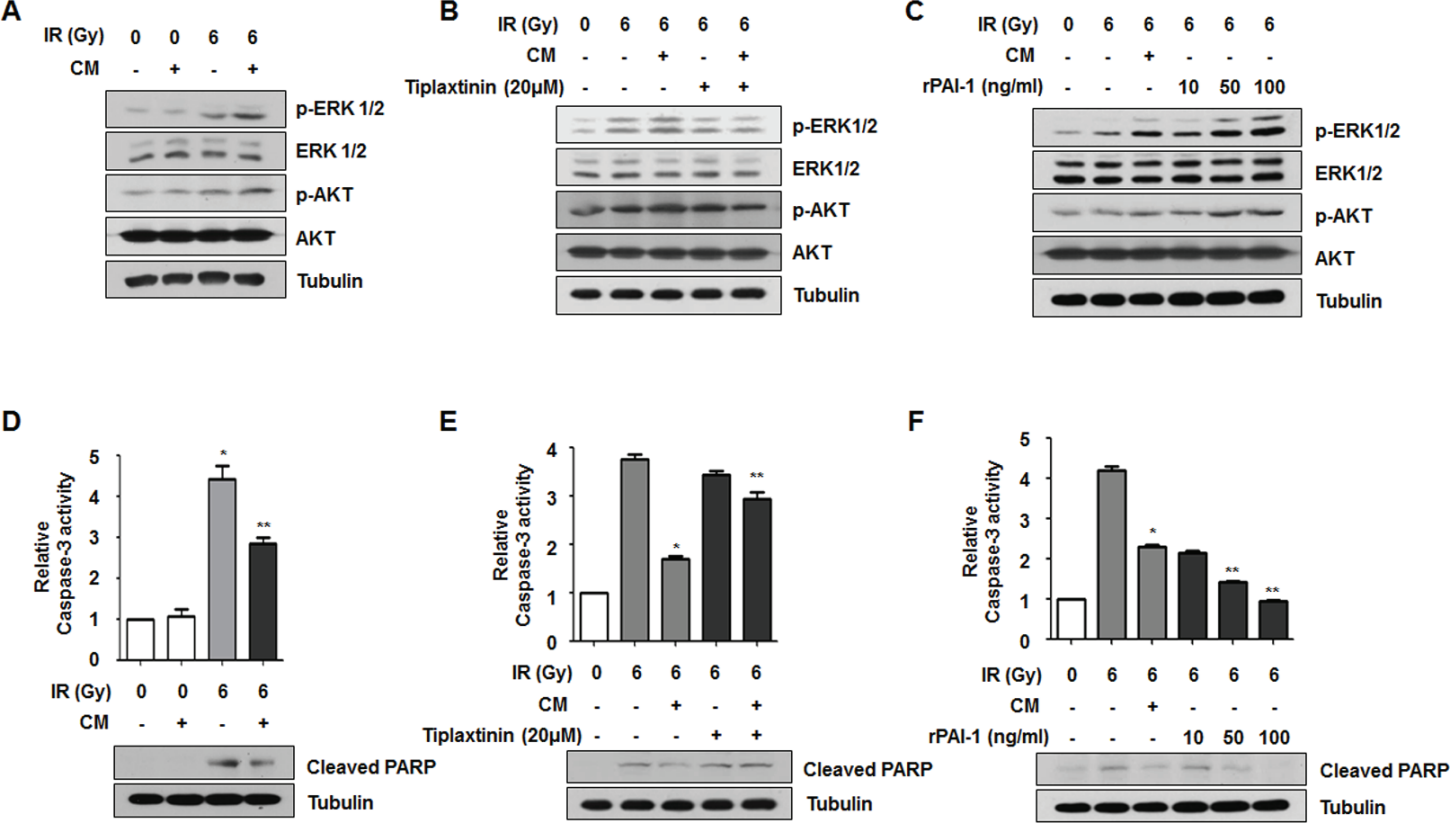
Secreted extracellular PAI-1 increases radioresistance of NCI-H460 cells through AKT and ERK1/2 activation and caspase-3 inhibition (**A**) Effects of PAI-1 secreted from A549 cells on activation of AKT and ERK1/2 signaling in NCI-H460 cells were analyzed by Western blotting. (**B** and **C**) Effects of PAI-1 on activation of AKT and ERK1/2 signaling in NCI-H460 cells were confirmed using a PAI-1-specific inhibitor or rPAI-1. (**D**) Effects of PAI-1 on activity of caspase-3 and PARP cleavage in NCI-H460 cells were analyzed by a Caspase-Glo^®^ 3/7 assay and Western blotting. *p < 0.05 compared with non-irradiated cells; **p < 0.05 compared with irradiated cells treated with control media. (**E** and **F**) The effects of PAI-1 on activity of caspase-3 and PARP cleavage in NCI-H460 cells were confirmed using a PAI-1-specific inhibitor (tiplaxtinin, 20 μM) or rPAI-1 (50 ng/ml). *p < 0.05 compared with irradiated cells treated with control media; **p < 0.05 compared with irradiated cells treated with CM.

Although several studies have investigated functional end-points of PAI-1 [28, 29], the precise downstream signaling of extracellular PAI-1 has not been clearly elucidated. Nevertheless, some studies have suggested that PAI-1 is involved in cell proliferation signaling through PI3K/AKT pathway and also induces phosphorylation of ERK1/2 and suppression of caspase-3 activity to stimulate cell survival signaling [12, 17–19]. Since activation of AKT, ERK1/2 and caspase-3 is critically associated with cell survival and death determining radiosensitivity, we assessed whether PAI-1 could lead to AKT and ERK1/2 activation in NCI-H460 cells. As shown in Figure 3A, the IR-induced increase of phosphorylated AKT and ERK1/2 was intensified by treatment with CM. To determine whether the effects of CM on phosphorylations of AKT and ERK1/2 are directly related to PAI-1 expression, NCI-H460 cells were treated with tiplaxtinin or rPAI-1. It is well-known that a small molecule inhibitor, tiplaxtinin, effectively inhibits PAI-1 activity by blocking integrity of the PAI-1-uPA-uPAR complex [30]. Highly increased phosphorylations of AKT and ERK1/2 following radiation and CM treatment was diminished by treatment of NCI-H460 cells with tiplaxtinin (Figure 3B). In contrast, phosphorylations of AKT and ERK1/2 in NCI-H460 cells in response to radiation were increased by treatment with rPAI-1 (Figure 3C). These results suggest that extracellular PAI-1 in CM enhances cell survival signaling by promoting the phosphorylations of ATK and ERK1/2. We next determined whether PAI-1 could inhibit apoptosis signaling to render radioresistance to radiosensitive cells. When NCI-H460 cells were exposed to CM of A549 cells, radiation-induced caspase-3 activation and subsequent PARP cleavage in NCI-H460 cells were significantly decreased relative to the IR-only treated group (Figure 3D). Such CM-reduced caspase-3 activity and PARP cleavage in NCI-H460 cells was recovered by administration of CM with tiplaxtinin (Figure 3E). Moreover, the levels of caspase-3 activity and PARP cleavage were markedly decreased by treatment with rPAI-1 (Figure 3F). Taken together, these results demonstrated that PAI-1 intensified radioresistance of nearby radiosensitive cells through activation of AKT and ERK1/2 signaling and suppression of caspase-3-mediated apoptosis.

### Secreted extracellular PAI-1 stimulates downstream signaling via extracellular complex formation, not clathrin-mediated endocytosis

**Figure 4:**
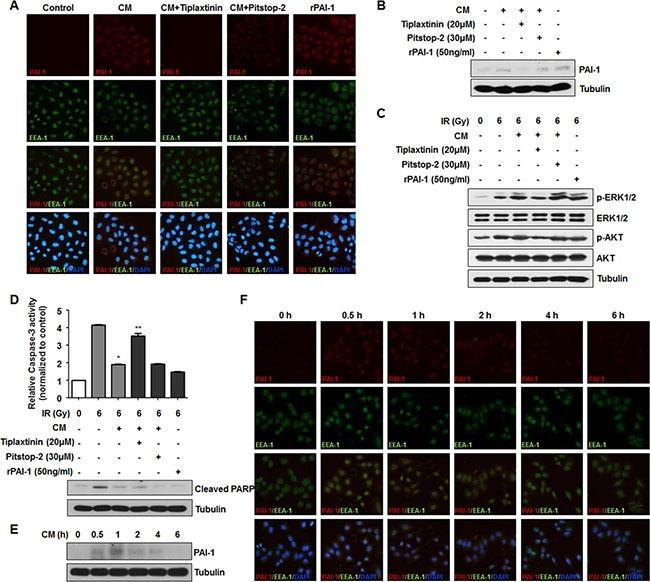
Secreted extracellular PAI-1 stimulates downstream signaling in NCI-H460 cells by extracellular interaction, not by clathrin-mediated endocytosis (**A**) Internalized PAI-1 in NCI-H460 cells after treatment with CM was analyzed by immunofluorescence. PAI-1, EEA-1, and cell nuclei are shown as red, green, and blue (DAPI) signals, respectively. PAI-1 endocytosed by clathrin-coated early endosome is indicated by co-stained red/green signals as yellow. (**B**) Intracellular levels of PAI-1 in NCI-H460 cells after treatment with CM were confirmed by Western blotting. (**C**) Effects of inhibition of PAI-1 endocytosis on AKT and ERK1/2 phosphorylation in NCI-H460 cells was analyzed by using tiplaxtinin (20 μM) or pitstop-2 (30 μM). (**D**) Effects of inhibition of PAI-1 endocytosis on activity of caspase-3 and PARP cleavage in NCI-H460 cells were analyzed using tiplaxtinin or pitstop-2. *p < 0.05 compared with irradiated cells treated with control media; **p < 0.05 compared with irradiated cells treated with CM. (**E** and **F**) Degradation of PAI-1 levels after endocytosis into NCI-H460 cells was analyzed by Western blotting and immunofluorescence, respectively. After treatment with CM from irradiated A549 cells, intracellular PAI-1 levels in NCI-H460 cells were measured from 0.5 h to 6 h.

Previous studies have suggested several mechanisms for extracellular PAI-1 effects on target cells. First, PAI-1 can bind to uPA, which specifically binds to uPAR. In this case, PAI-1 easily binds to low density lipoprotein receptor-related protein 1 (LRP-1) and is subsequently endocytosed in a clathrin dependent manner [31]. Second, PAI-1 can bind to vitronectin, then inhibit cell adhesion [32]. Third, intracellular PAI-1 can interact with other factors, such as caspase-3, leading to its inactivation and promoting an anti-apoptotic effect, while uPAR and LRP-1 are recycled to the cell surface [18]. To clearly determine how extracellular PAI-1 mediates intracellular downstream signaling of nearby radiosensitive cells, NCI-H460 cells were treated with two types of inhibitors, tiplaxtinin for PAI-1 inhibition and pitstop-2 to block formation of endocytosis pits. We observed that after treatment of CM from A549 cells or rPAI-1, PAI-1 is significantly introduced into NCI-H460 cells as indicated by co-staining with early endosome antigen 1 (EEA-1) and PAI-1 (Figure 4A). When NCI-H460 cells were treated with tiplaxtinin or pitstop-2, the endocytosis of NCI-H460 cells by PAI-1 was blocked. The level of intracellular PAI-1 in NCI-H460 cells after CM treatment was also confirmed by Western blotting (Figure 4B). We further investigated whether treatment with both inhibitors could alleviate PAI-1-mediated downstream signaling. Interestingly, treatment with pitstop-2 did not reduce the increase of AKT and ERK1/2 phosphorylation increased by CM treatment, while administration of tiplaxtinin reduced AKT and ERK1/2 phosphorylation (Figure 4C). Furthermore, the levels of activated caspase-3 and PARP cleavage were recovered by tiplaxtinin treatment, but not by pitstop-2 treatment (Figure 4D). To demonstrate whether PAI-1 introduced via endocytosis could directly mediate intracellular signaling, the level of intracellular PAI-1 was monitored over time. The level of endocytosed PAI-1 in NCI-H460 cells peaked at 30 min and 1 h after CM treatment, then decreased gradually with time via endosomal degradation (Figure 4E, 4F). Taken together, these results suggest that radioresistant signaling in radiosensitive cells was mediated through complex formation of extracellular PAI-1 with its partners, not through clathrin-mediated endocytosis.

### Secreted extracellular PAI-1 reinforces radiation-induced EMT of radiosensitive cells in a paracrine manner

**Figure 5:**
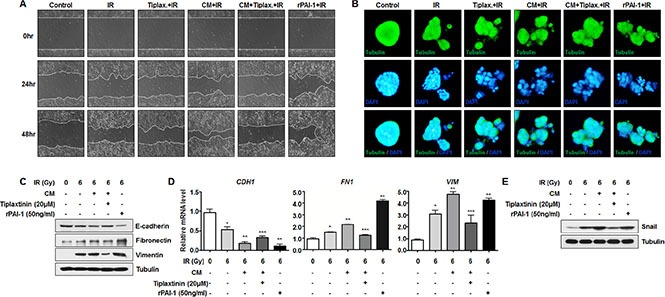
Secreted extracellular PAI-1 enhances radiation-induced EMT in radiosensitive NCI-H460 cells (**A**) Effects of PAI-1 on radiation-induced migration in NCI-H460 cells were analyzed by a wound-healing assay. (**B**) Effects of PAI-1 on morphological changes in NCI-H460 cells were monitored with a 3D culture model. The cells were then permeabilized and stained for tubulin (green) and with DAPI (blue). (**C**) Effects of PAI-1 on protein expression of E-cadherin, Fibronectin, and Vimentin in NCI-H460 cells were analyzed by Western blotting. (**D**) Effects of PAI-1 on mRNA expression of *CDH1*, *FN1*, and *VIM* in NCI-H460 cells were analyzed by real time qRT-PCR. *p < 0.05 compared with non-irradiated cells; **p < 0.05 compared with irradiated cells treated with control media; ^***^p < 0.05 compared with irradiated cells treated with CM. (**E**) The effects of PAI-1 on protein expression of Snail in NCI-H460 cells were analyzed by Western blotting.

A recent report suggested that PAI-1 could also induce cancer invasion and metastasis by promoting EMT via interacting with integrin, LRP-1, uPA-uPAR, and the ECM, or via indirect classical signaling pathways [33]. Moreover, our previous studies revealed that radiation could increase migration capacity of NSCLC cells and expression of EMT markers [34–36]. To examine the effects of secreted PAI-1 on the aggressiveness of radiosensitive cells, we measured the migration capacity and expression levels of EMT markers in NCI-H460 cells. Wound healing assay revealed that cell motility increased when NCI-H460 cells were treated with CM and irradiation, compared to groups treated with irradiation-alone or groups treated with irradiation and tiplaxtinin (Figure 5A). In addition, increased motility of NCI-H460 cells decreased significantly when cells were incubated with CM containing tiplaxtinin, while treatment with rPAI-1 dramatically increased cell migration (Figure 5A). To monitor the morphological changes in NCI-H460 cells without perturbation of normal cancer growth architecture, NCI-H460 cells were cultured on a thick layer of matrigel to form epithelial acini. In the same context as the wound healing assay, NCI-H460 cells treated with CM and radiation were distributed more widely. In addition, their motility was reduced by treatment with tiplaxtinin and increased by treatment with rPAI-1 (Figure 5B). Moreover, CM treatment accelerated radiation-induced EMT by decreasing the expression of E-cadherin (an epithelial phenotype marker) while increasing the expression of Fibronectin and Vimentin (two mesenchymal markers) at both the protein and mRNA levels in NCI-H460 cells (Figure 5C, 5D). Promoted EMT in NCI-H460 cells, as indicated by a reduced level of E-cadherin and increased levels of Fibronectin and Vimentin, was also reduced by treatment with tiplaxtinin and markedly increased by treatment with rPAI-1. According to a previous study, PAI-1 also increases expression of Snail, which is a transcriptional regulator responsible for downregulation of E-cadherin [37]. To clarify how secreted PAI-1 promotes the expression of EMT markers, we investigated whether extracellular PAI-1 could increase the expression of Snail. As shown in Figure 5E, Snail expression in NCI-H460 cells in response to radiation was further increased by CM treatment. This increased level of Snail was reduced by tiplaxtinin treatment and increased by treatment with rPAI-1. In conclusion, these results suggest that PAI-1 secreted from radioresistant cells could facilitate EMT of nearby radiosensitive cells and make them more aggressive and resistant to radiotherapy.

### Inhibition of PAI-1-mediated intercellular communication increases *in vivo* radiosensitization and decreases *in vivo* EMT in a xenograft mouse model

**Figure 6:**
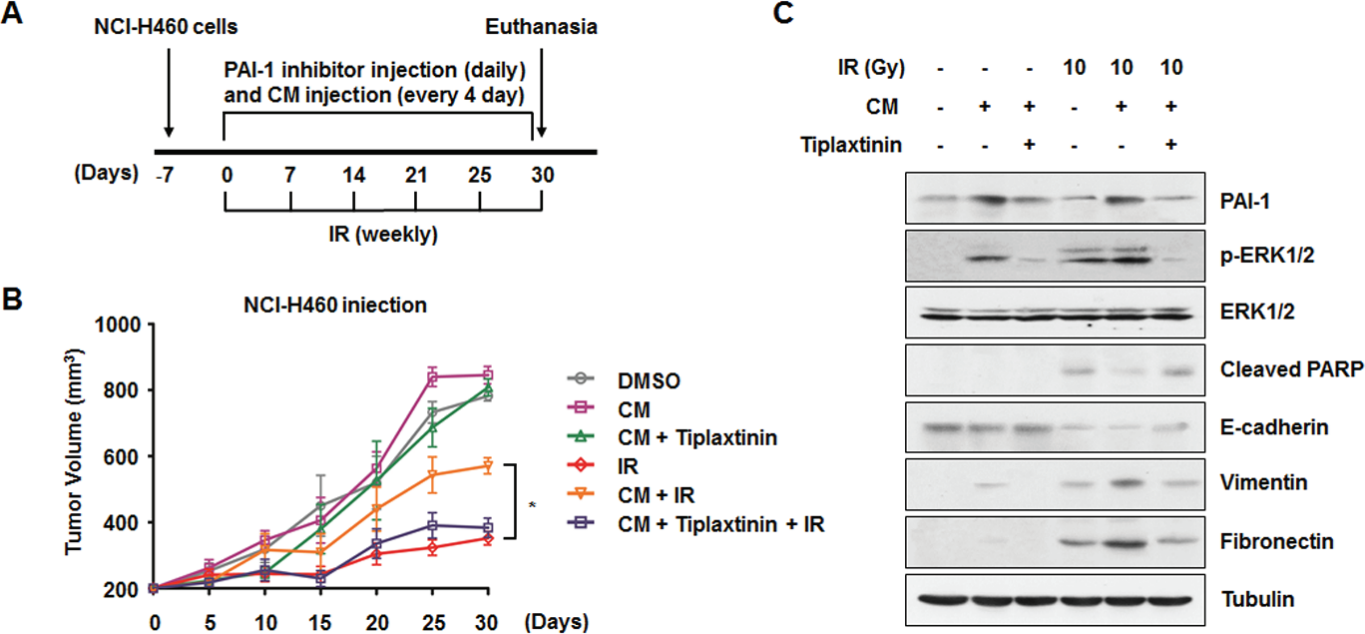
Inhibition of PAI-1-mediated intercellular communication decrease *in vivo* radioresistance and EMT in a xenograft mouse model (**A**) An experimental protocol to determine whether a PAI-1 inhibitor decreases *in vivo* radioresistance and EMT in a xenograft mouse model. (**B**) Effects of CM of A549 cells and a PAI-1 inhibitor on *in vivo* radioresistance in a xenograft mouse model. *p < 0.05 for tumor tissues from irradiated mice treated with CM versus tumor tissues from animals treated with radiation alone or those treated with radiation, CM, and tiplaxtinin. (**C**) *In vivo* effects of CM and a PAI-1 inhibitor on ERK1/2 phosphorylation, PARP cleavage, and expression of EMT-related proteins were evaluated by Western blotting.

As described above, we found that secreted PAI-1 could increase cell survival after irradiation and promote IR-induced EMT in NCI-H460 cells (Figures 1, 5). To evaluate the effects of PAI-1 on radiosensitivity and progression of lung tumor *in vivo*, a xenograft mouse model was established (Figure 6A). *In vivo* data from nude mice bearing tumors formed by NCI-H460 cells indicated that PAI-1-containing CM could also make tumors more resistant to radiation in parallel with *in vitro* data (Figure 6B). Tumor volumes of mice treated with CM and radiation were significantly increased by approximately 2 fold on day 30 when compared with mice that received radiation alone. In addition, the effects of extracellular PAI-1 on acquisition of radioresistance were offset by functional inhibition of PAI-1 with tiplaxtitnin treatment. Consistent with our results *in vitro* (Figures 3, 5), ERK1/2 phosphorylation was increased and PARP cleavage was decreased in the extracted tumor tissue lysates when CM was administered to mice. Furthermore, decrease of E-cadherin and increase of Vimentin and Fibronectin were promoted by CM treatment relative to the radiation only group. In all experiments, PAI-1-mediated expression of marker proteins was recovered by treatment with tiplaxtinin (Figure 6C). Overall, these data suggest that the functional inhibition of secreted PAI-1 with treatment of a specific inhibitor could significantly sensitize lung tumors to radiation *in vivo* while suppressing the EMT.

## DISCUSSION

Modern radiotherapy is generally administered at optimal time intervals to minimize damaging effects to adjacent normal cells and tissues of tumor mass. However, this therapeutic procedure results in radioresistance of tumor cells, which acts as a fundamental barrier limiting the effective treatment and contributing to the repopulation of tumor cells during anti-cancer therapies. In a tumor, various subclonal tumor cells with distinct characteristics and genetic variation exist, and they can differentially respond to extracellular stress signals such as irradiation and cytotoxic drugs [4–6]. Several studies have reported the involvement of tumor microenvironments and secretory factors responsible for treatment failure. Paracrine factors, including interleukin-17, WNT16B, and CXCL1, secreted from immune cells and cancer-associated fibroblasts have been investigated as key molecules making the nearby tumor cells (so called ‘recipient cells’) more impervious to chemotherapies [10, 11, 38]. Considering the intratumoral genetic heterogeneity, we focused on identification of paracrine factors derived from tumor cells capable of helping surrounding tumor cells be aggressive and therapy-resistant in response to radiation. We identified PAI-1 as a major factor that turns radiosensitive cells into radioresistant cells in a paracrine manner, via a cell-extrinsic action that enhances the proliferation of recipient PAI-1-negative/low NSCLC cells through PAI-1-mediated AKT and ERK1/2 activation. Our findings suggest that PAI-1 can be a potent target as a paracrine factor for survival and EMT induction correlated with radioresistance in radiosensitive NSCLC cells.

PAI-1 has been reported to promote anti-apoptosis signaling through inhibition of caspase-3 [39–41]. As shown in Figures 3 and 4, we found that PAI-1 secreted from A549 cells inhibited caspase-3 activity and subsequent PARP cleavage in NCI-H460 cells. Our data and the previous studies, nevertheless, did not provide direct evidence for PAI-1 induced caspase-3 inhibition. We could not exclude the possibility that caspase-3 activation is suppressed indirectly by the outcome of cell survival signaling and cell viability. Further studies for biochemical analysis to investigate the direct relationship between PAI-1 and caspase-3 would be required.

Although there have been controversial roles of PAI-1 in tumor reported, accumulated evidence suggests that it is highly involved in malignant progression and poor prognosis in metastatic tumors. High levels of PAI-1 expression have been reported to be significantly correlated with node metastasis and a short disease-free survival in patients with several types of cancer including, HNSCC and NSCLC [15, 42]. In the current study, we showed that, when exposed to high levels of PAI-1, PAI-1-negative cells are capable of promoting cell survival and EMT against irradiation, leading to therapy-resistant properties. One important finding of our study is that radiosensitive cancer cells can become radioresistant through cell-cell communication by PAI-1, which may be secreted in response to radiation from other cancer cells possessing radioresistant properties. Thus, the increase of PAI-1 levels in a paracrine manner through interactions of both cell-cell and cell-environments may synergistically influence therapeutic sensitivity. Our findings are supported by a study demonstrating that tumor progression and angiogenesis of PAI-1-positive tumor cells after subcutaneous implantation was observed in wild-type mice, but not PAI-1-deficient mice [43]. These findings implied that the paracrine signaling of PAI-1 in implanted tumor cells provided by tumor surroundings consisting of host cells is critical to promotion of tumor progression.

Highly advanced tumor cells have been reported to adapt to harsh tumor microenvironments such as chronic hypoxia and ROS stress to evade cell death [44], and pre-established hypoxic environments in tumors can function as barriers to decreasing radiotherapeutic efficacy [45]. We observed that these stresses such as hypoxia, excessive ROS levels, and radiation can act as stimulants for PAI-1 expression and secretion. It has been shown that PAI-1 may, at least in part, contribute to adaptation to stress-induced responses according to its roles in anti-apoptotic and radioresistance-promoting functions. In addition, PAI-1 is involved in induction of angiogenesis [39, 43]. Considering the correlation of angiogenesis with metastasis and their contributions to poor clinical outcomes after radiotherapy, we focused on a role of PAI-1 in metastatic conversion of tumor cells. We found that PAI-1 participates in EMT induction through regulation of epithelial- and mesenchymal-associated protein expression (Figure 5). Our findings and those of previous studies suggest that PAI-1 can be a potent marker of poor prognosis and restriction to low PAI-1 levels at tumor sites, which will be helpful for positive therapeutic efficacy.

After secretion from a cell, PAI-1 mediates intracellular signaling in target cells through extracellular binding to specific receptors such as LRP-1. Since all members of the LRP family have been reported to be capable of receptor-mediated endocytosis mainly in a clathrin-dependent manner, they can undergo endosomal trafficking for either receptor recycling or lysosomal degradation after transducing ligand-mediated signaling [46–48]. In addition, the receptors can be internalized with their ligands in early endosomes to provide a signaling-mediating platform for additional signal-responses as proposed in the signaling endosome hypothesis [49]. In the current study, we found that PAI-1 is introduced into early endosomes of radiosensitive NSCLC cells via clathrin-mediated endocytosis which was confirmed by treatment with pitstop-2, and the residual amount of PAI-1 diminished in a time dependent manner (Figure 4). Based on the rapid decrease of PAI-1 levels (within a few hours) in radiosensitive NSCLC cells, we assumed that the signaling turnover conducted by the complex of PAI-1 and its receptor might be short-lived due to lysosomal degradation, not by acting as a signal-platform to sustain extra-responses in endosomes. In addition, PAI-1 might mediate radioresistance signaling through extracellular interaction with its receptor regardless of endocytosis. We found that treatment with tiplaxtinin counteracted CM- or rPAI-1-induced AKT and ERK1/2 phosphorylation and caspase-3 inhibition, but that pitstop-2 did not. According to the action mechanisms of each inhibitor, these data indicated that interaction of PAI-1 with its receptor at the cell surface is necessary to transduce therapy-resistance signaling. Conclusively, our findings suggest that PAI-1 can be a promising therapeutic target and satisfactory outcomes from radiotherapy may be achieved by the systemic administration of a specific PAI-1 inhibitor including tiplaxtinin as a radioadjuvant agent.

**Figure 7:**
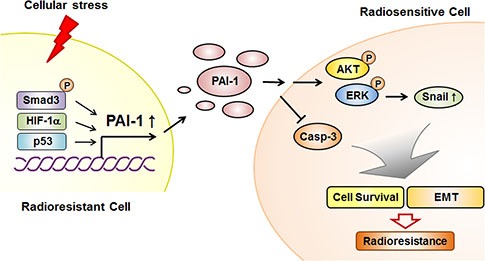
Schematic diagram illustrating that PAI-1 secreted from radioresistant NSCLC cells induces paracrine signaling in radiosensitive NSCLC cells, leading to increased survival capacity and EMT phenotype In radioresistant NSCLC cells, PAI-1 is increased in response to cellular stresses such as irradiation, leading to increased extracellular PAI-1. The increased level of extracellular PAI-1 can interact with its receptor at the surface of radiosensitive NSCLC cells. After binding of PAI-1 to its receptor in radiosensitive NSCLC cells, cell survival and proliferation are induced by PAI-1-mediated AKT and ERK1/2 phosphorylation and caspase-3 inactivation. In addition, extracellular PAI-1 promotes EMT by regulating expression levels of EMT-related proteins, including Snail. This intercellular communication mediated by PAI-1 makes radiosensitive NSCLC cells more resistant to radiation.

The exact molecular mechanism governing intratumor-radioresistance in NSCLC has been unclear to date. In this study, we provided evidence for the function of PAI-1 as a paracrine factor inducing radioresistance and demonstrated the mechanism of PAI-1-mediated radioresistance in NSCLC under irradiation. The results presented herein define this radioresistance mechanism through functional orchestration of PAI-1 and AKT/ERK/Snail axis in NSCLC cells and provide a possible explanation for how NSCLC could acquire and intensify intratumor radioresistance (Figure 7). In addition, we demonstrated mechanisms of EMT and migration of radiosensitive NSCLC cells after binding of secreted-PAI-1. Although we did not investigate a cohort of patients with NSCLC, the results of our study demonstrate that the targeting of secreted PAI-1 in combination with radiotherapy could overcome radioresistance and eventually enhance the efficacy of radiotherapy for treatment of NSCLC.

## MATERIALS AND METHODS

### Cell lines, cell culture, irradiation, and drug treatment

The human NSCLC cell lines, A549, NCI-H460, NCI-H292, and NCI-H358, were acquired from the *American Type Culture Collection* (ATCC, Manassas, VA), authenticated, and maintained in early passages, no more than 6 months after receipt from ATCC. Cells were grown in RPMI-1640 medium supplemented with 10% FBS, 100 U/mL penicillin, and 100 mg/mL streptomycin at 37°C in 95% air/5% CO_2_. Cells were exposed to a single dose of γ-rays using a Gamma Cell 40 Exactor (Nordion International, Inc., Kanata, Ontario, Canada) at a dose rate of 0.81 Gy/min. Flasks containing control cells were placed in the irradiation chamber, but not exposed to radiation. Cells were treated with tiplaxtinin dissolved in dimethyl sulfoxide (DMSO) and recombinant PAI-1 dissolved in 10 mM sodium phosphate for 6 h before irradiation.

### Preparation of conditioned media

Cells were plated at a density of 5 × 10^4^ cells/mL in 100-mm culture dishes, incubated for 24 h, and then exposed to 6 Gy of IR. At 2.5 days after irradiation, cells were washed with PBS three times, then further incubated in serum-free media without antibiotics for 36 h. CM were collected and centrifuged to remove any residual cells, after which they were filtered through a 0.2 μm syringe filter. Filtered CM was concentrated 10-fold using a Centricon-10 concentrator (Millipore, Billerica, MA) at 4°C, then stored at −20°C. Following CM collection, the number of cells on the dish was determined and the volume of CM used in each experiment was normalized for cell number.

### Colony forming assay

A colony forming assay was performed as previously described [36]. Briefly, cells were plated at a density of 300 cells per well in six-well dishes. After 24 h, cells were treated with the indicated drugs or exposed to a specific dose of radiation and then allowed to grow for 7 d. Next, the cells were fixed with 10% methanol and 10% acetic acid, after which they were stained with 1% crystal violet. Colonies containing more than 50 cells were identified using densitometry software and scored as survivors.

### Apoptosis assay

An Annexin V-FITC kit (Enzo Life Science, Farmingdale, NY) was used to detect apoptosis as previously described [50]. Following the treatment of CM from NSCLC cells lines and 6 Gy of irradiation, cells (10^5^−10^6^ cells/mL) were harvested, washed with ice-cold PBS, and resuspended in 100 mL of ice-cold 1× binding buffer. Next, 25 ng of Annexin V-FITC and 250 ng of propidium iodide were added to the cell suspension and the cells were incubated on ice for 10 min in the dark. Finally, the stained cells were diluted to a final volume of 250 mL with 1× binding buffer and analyzed using a FACSVerse flow cytometer (BD Biosciences, San Jose, CA).

### Caspase-3 activity assay

A Caspase-Glo 3/7 assay kit (Promega, Madison, WI) was used to measure caspase-3/7 activities as previously described [50]. Briefly, cells (2 × 10^4^ cells/well) were seeded overnight in a 96-well plate, then incubated with the desired treatment of CM or irradiation. Subsequently, 100 μL of Caspase-Glo 3/7 reagent containing caspase-3/7 substrate was added to each well. After the contents of the wells were gently mixed at 300–500 rpm for 30 sec, the plate was incubated at room temperature for 2 h. Finally, the luminescence of each sample was measured using a Glomax multi detection system (Promega).

### Wound healing assay

A wound healing assay was performed to measure changes in cell motility as previously described [35]. Briefly, cells were cultured to 70% confluency and treated with CM, tiplaxtinin, or rPAI-1. The cell monolayers were then scratched with a 200 μL pipette tip, after which NCI-H460 cells were further incubated with fresh medium with or without irradiation for 48 h. Photomicrographs were then taken at 100× magnification using an Olympus IX71 inverted microscope (Olympus Optical Co. Ltd., Tokyo, Japan).

### Tumor xenografts in nude mice

Tumor xenografts in nude mice were conducted as previously described [51]. Six-week-old male BALB/c athymic nude mice (Central Lab Animals Inc., Seoul, South Korea) were used for the *in vivo* experiments. The animals (*n* = three per group) were injected with normal media or CM-adapted 2 × 10^6^ NCI-H460 cells in the flank and tumors were allowed to develop. Upon identification of a palpable tumor (minimum volume of 200 mm^3^), DMSO or PAI-1 inhibitor (tiplaxtinin, 200 μg/kg body weight) was administered via oral gavage daily for 30 days. The CM was then injected into the tumors using insulin syringes every four days. Mice in non-CM treated groups were treated with normal media as a control. The animals were also irradiated with 10 Gy once a week for 4 weeks. Tumor length (L) and width (l) were measured with a caliper and tumor volumes were calculated using the formula (L × l^2^)/2. At the end of the treatment period, animals were euthanized and the tumors were used for biochemical studies. Animal care protocol is detailed in Supplementary Materials and Methods.

### Statistical analysis

All numeric data are presented as the means ± standard deviation (SD) from at least three independent experiments. Experimental results were analyzed by one-way ANOVA for ranked data followed by Tukey's honestly significant difference test, and two-way ANOVA for ranked data followed by the Bonferroni post hoc test. The Prism 5 software (GraphPad Software, SanDiego, CA) was used to conduct all statistical analyses. A *p*-value < 0.05 was considered to be statistically significant.

## SUPPLEMENTARY MATERIALS FIGURES AND TABLE


