# Supplementary material for: Plasminogen activator inhibitor-1 enhances radioresistance and aggressiveness of non-small cell lung cancer cells
Source: Oncotarget. 2016 Mar 19;7(17):23961–74. doi: 10.18632/oncotarget.8208 (PMC5029677; doi:10.18632/oncotarget.8208)
Supplement: Supplementary file 1 [file oncotarget-07-23961-s001.pdf]

Plasminogen activator inhibitor-1 enhances radioresistance and aggressiveness of non-small cell lung cancer cells

Supplementary Materials

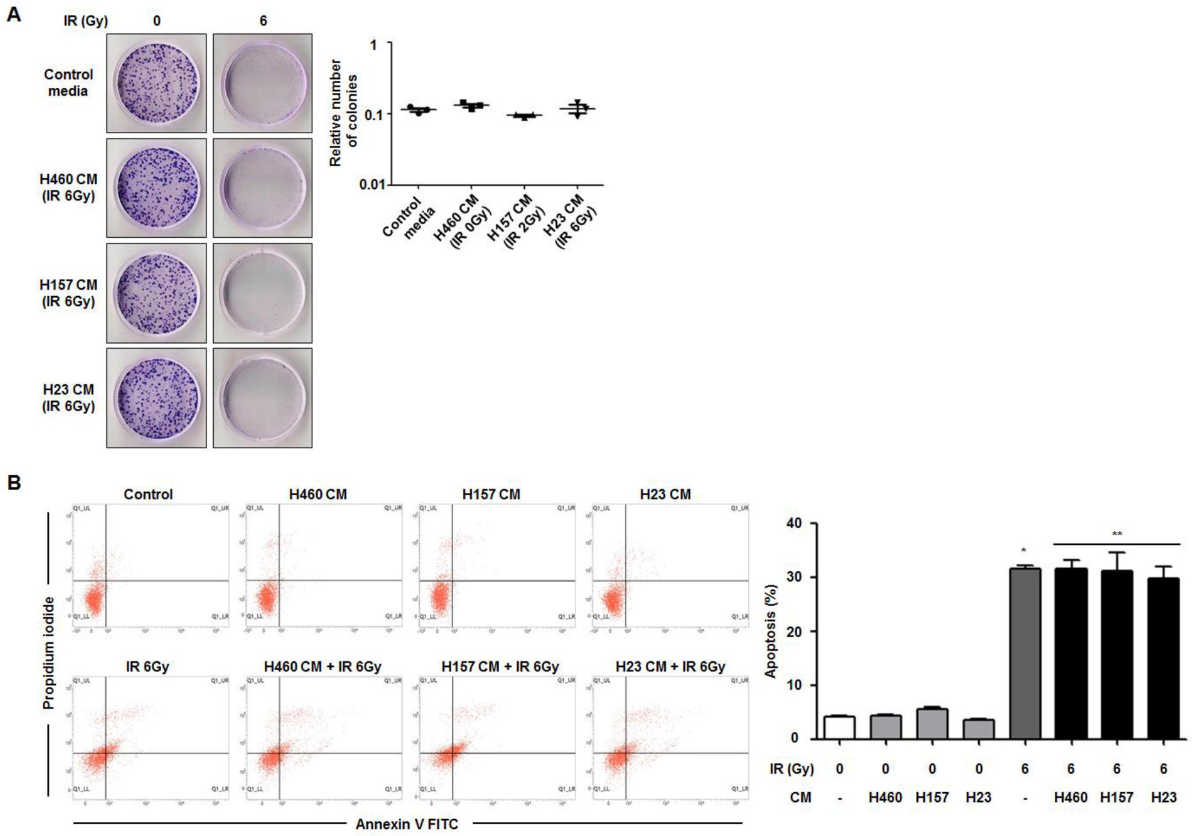

Supplementary Figure S1: Secretome from radiosensitive cells has no effects on survival of NCI-H460 cells. **(A)** Effects of CM derived from radiosensitive cell lines, NCI-H460, NCI-H157, and NCI-H292 cells, on survival of NCI-H460 cells in response to radiation were measured by a colony forming assay. **(B)** Effects of CM derived from NCI-H460, NCI-H157, and NCI-H292 cells on radiation-induced apoptosis of NCI-H460 cells were analyzed by an Annexin V staining assay. The relative levels of Annexin V- and PI-positive populations of NCI-H460 cells are indicated in the graph. $^{*}p < 0.05$ compared with non-irradiated cells; $^{**}p > 0.05$ compared with irradiated cells treated with negative control media.

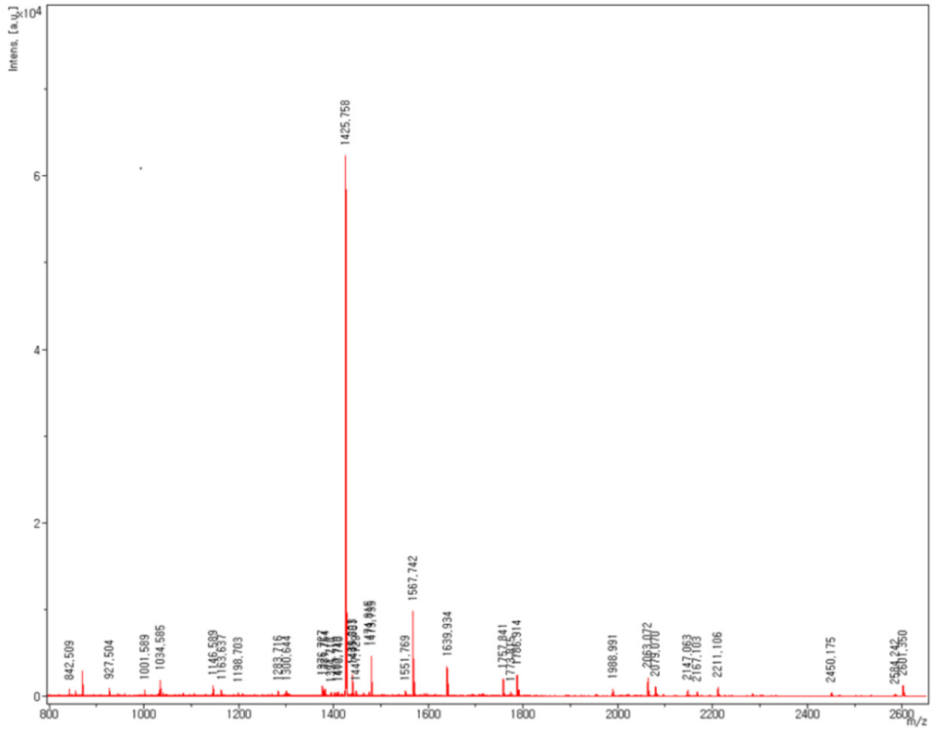

Supplementary Figure S2: Peptide mass fingerprinting map. Protein band described at Figure 2A was excised, digested with trypsin and subjected to MALDI-TOF analysis (Microflex LRF20). Spectra were collected and calibrated using Trypsin auto-digestion peaks (m/z 842.5099, 2211.1046).

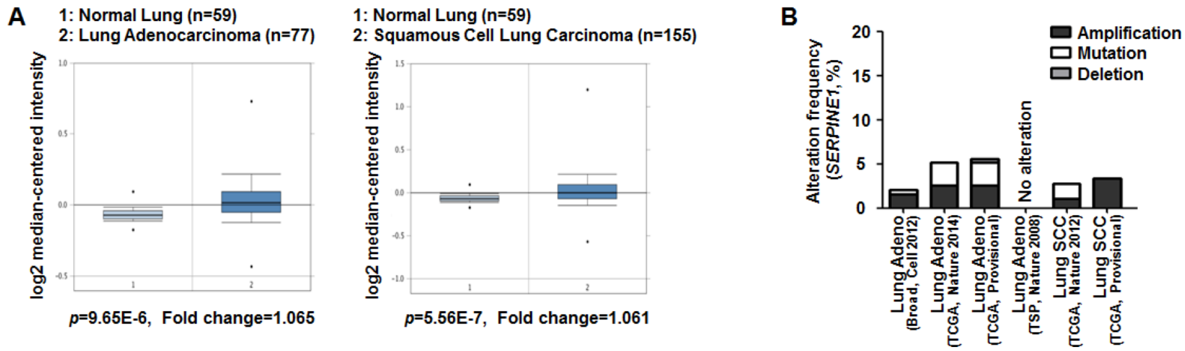

Supplementary Figure S3: Lung tumors did not show significant induction of PAI-1 expression. (**A**) Datasets available from Oncomine (http://www.oncomine.org) demonstrated that lung adenocarcinomas and squamous cell lung carcinomas expressed similar levels of *SERPINE1* to normal lung (Ref#23; [Left] $p = 9.65 \times 10^{-6}$, fold change = 1.065; [Right] $p = 5.56 \times 10^{-7}$, fold change = 1.061). (**B**) Datasets obtained from cBioportal (http://www.cbioportal.org) demonstrated that genetic alterations of *SERPINE1* were present, but rare in NSCLCs (Ref#24, 25).

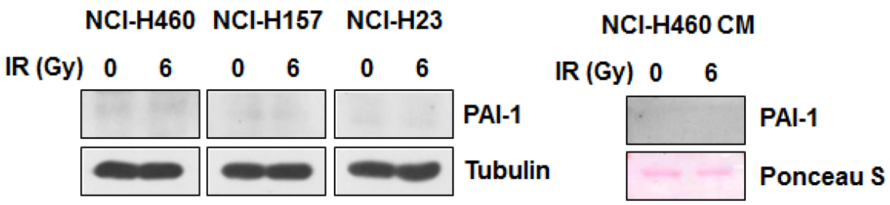

Supplementary Figure S4: Expression levels of PAI-1 in radiosensitive NSCLC cells in response to radiation. Radiation-induced PAI-1 expression in NCI-H460, NCI-H157, and NCI-H23 cells and CM of NCI-H460 cells were analyzed by Western blotting.

MATERIALS AND METHODS

Chemicals, antibodies, and reagents

Tiplaxtinin, pitstop-2, and recombinant PAI-1 (rPAI-1) were obtained from Axon Medchem (Reston, VA), Abcam (Cambridge, MA), and GenWay (San Diego, CA), respectively. Antibodies specific for PAI-1 (sc-5297), Tubulin (sc-5546), p53 (sc-126), HIF-1α (sc-53546), Snail (sc-28199), E-cadherin (sc-7870), Vimentin (sc-32322), and Fibronectin (sc-9068) purchased from Santa Cruz Biotechnology (Santa Cruz, CA), phosphorylated (p)-Smad3 (9520), AKT (9272), p-AKT (9271), ERK1/2 (9102), p-ERK1/2 (9101), and PARP (9542) purchased from Cell Signaling Technology (Beverly, MA), and EEA-1 (ab2900) purchased from Abcam were used for Western blotting or immunofluorescence. Cell culture medium (RPMI-1640), FBS, glutamine, penicillin, and streptomycin were acquired from Gibco (Grand Island, NY). Control siRNA and siRNA specific for PAI-1 #1 (ON-TARGETplus SMARTpool) were obtained from Dharmacon (Chicago, IL), and PAI-1 siRNA #2 (AccuTarget™ siRNA) was obtained from Bioneer (Daejeon, Republic of Korea).

Silver staining and peptide mass fingerprint

For identification of novel candidates, the conditioned media were subjected to SDS-PAGE and silver stained using Pierce Silver Stain for Mass Spectrometry (Thermo Scientific, Rockford, IL) according to the manufacturer's instructions. Subsequently, the stained gel was analyzed by Peptide Mass Fingerprint (PMF) including MALDI-TOF at Genomine Inc (Pohang, South Korea). For protein identification by peptide mass fingerprinting, protein spots were excised, digested with trypsin (Promega, Madison, WI), mixed with α cyano-4-hydroxycinnamic acid in 50% acetonitrile/0.1% TFA, and subjected to MALDI-TOF analysis (Microflex LRF 20, Bruker Daltonics, Breman, Germany) as described by Fernandez *et al.* [1] Spectra were collected from 300 shots per spectrum over an m/z range of 600–3000 and calibrated by two point internal calibration using Trypsin auto-digestion peaks (m/z 842.5099, 2211.1046). A peak list was generated using Flex Analysis 3.0. The threshold used for selection of peaks was as follows: 500 for minimum resolution of monoisotopic mass, 5 for S/N. The MASCOT search program developed by Matrixscience (http://www.matrixscience.com/) was used for protein identification by peptide mass fingerprinting. The following parameters were used for the database search: trypsin as the cleaving enzyme, a maximum of one missed cleavage, iodoacetamide (Cys) as a complete modification, oxidation (Met) as a partial modification, monoisotopic masses, and a mass tolerance of ± 0.1 Da. PMF acceptance criteria was based on probability scoring.

Western blot analysis

Following the experimental treatments, Western blot analysis was performed as previously described [2]. Following the treatment of CM and 6Gy of irradiation, whole cell lysates (WCL) were prepared using radioimmunoprecipitation assay (RIPA) lysis buffer (50 mM Tris, pH 7.4, 150 mM NaCl, 1% Triton X-100, 25 mM NaF, 1 mM dithiothreitol (DTT), 20 mM EGTA, 1 mM Na_3VO_4, 0.3 mM phenylmethanesulfonyl fluoride (PMSF), and 5 U/ml aprotinin) and protein concentrations in the lysates were determined using a BioRad protein assay kit (BioRad Laboratories, Hercules, CA). Protein samples were subjected to SDS-PAGE, transferred to a nitrocellulose membrane and then blocked with 5% skim milk in TBST (10 mM Tris, 100 mM NaCl, and 0.1% Tween 20) for 1 h at room temperature. Next, membranes were probed with specific primary antibodies and peroxidase-conjugated secondary antibody (Santa Cruz Biotechnology). The samples were then using an ECL detection system (Roche Applied Science, Indianapolis, IN). Densitometric analysis was performed using the Scion Image software (Scion Corporation, Frederick, MD).

Immunofluorescence (IF) staining

IF staining studies were conducted as previously described [3]. Following the experimental treatments including CM from A549 cells, tiplaxtinin, Pitstop2, or rPAI-1, cells were fixed with 2% paraformaldehyde for 20 min, permeabilized in 0.5% Triton X-100 for 10 min, then washed with cold PBS. After blocking with 1% bovine serum albumin/PBS, the cells were incubated overnight with anti-PAI-1 or -EEA1 antibody at 4°C. Next, cells were washed three times with cold PBS and then incubated with DyLight 488- and DyLight 649-conjugated secondary antibodies (Thermo Scientific). After washing and counterstaining with 4′,6-diamidino-2-phenylindole (Sigma, St. Louis, MO), the glass slides were mounted with a Vectashield Hard-Set Mounting Medium (Vector Laboratories, Burlingame, CA) and visualized with an Olympus IX71 fluorescence microscope (Olympus Optical Co. Ltd., Tokyo, Japan).

Cell assay (3D culture)

A three-dimensional (3D) acini culture was conducted to detect morphological changes in the epithelial acini as previously described [4]. Matrigel (BD Biosciences) was thawed overnight at 4°C and mixed well using prechilled pipette tips before use. Cells were cultured on eight-well chambered glass slides (Nunc, Naperville, IL) with 100% growth factor-reduced Matrigel, and treated with CM, tiplaxtinin, or rPAI-1. The glass slides were then incubated at 37°C for at least 30 min, after which cells

were harvested, counted, and resuspended as a single-cell suspension of 25, 000 cells/mL in RPMI medium. Next, 200 µL of this cell suspension was mixed with 200 µL of RPMI medium containing 4% Matrigel, and the entire 400 µL was dispensed into each well of the glass slides. The cells were incubated and attached in a 37°C, 5% CO_2 incubator for 3 d. After the desired treatments, cells were incubated for an additional 28 h. Finally, the cells and acini were analyzed by IF staining using anti-Tubulin antibody (Santa Cruz Biotechnology).

Real-time quantitative RT-PCR (qRT-PCR)

The levels of EMT-related gene expression were measured by real-time qRT-PCR as previously described [5]. Aliquots of a master mix containing all of the reaction components with the primers (Table S1) were dispensed into a real time PCR plate (Applied Biosystems, Foster City, CA). All of the PCR reagents were from a SYBR Green core reagent kit (Applied Biosystems). The expression of all genes evaluated was measured in triplicate in the reaction plate. qRT-PCR was performed using an Applied Biosystems-7900 HT qRT-PCR instrument. PCR was performed by subjecting the samples to 15 s at 95°C and 1 min at 60°C for 40 cycles followed by thermal denaturation. The expression of each gene relative to *GAPDH* mRNA was determined using the $2^{-\Delta\Delta CT}$ method [6]. To simplify the data, values for the relative expression were multiplied by 10^2.

Animal protocol

Six-wk-old male BALB/c athymic nude mice (Central Lab Animals Inc., Seoul, South Korea) were used for the in vivo experiments. The animal protocols were approved by the Institutional Animal Care and Use Committee of Pusan National University (Busan, South Korea), and performed in accordance with the provisions of the NIH Guide for the Care and Use of Laboratory Animals. Mice were housed individually or in groups of up to five in sterile cages. Animals were maintained in animal care facilities in a temperature-regulated room (23 ± 1°C) under a 12 h light/dark cycle and quarantined for 1 wk prior to the study. The animals were fed water and a standard mouse chow diet ad libitum.

REFERENCES

1. Fernandez J, Gharahdaghi F, Mische SM. Routine identification of proteins from sodium dodecyl sulfate-polyacrylamide gel electrophoresis (SDS-PAGE) gels or polyvinyl difluoride membranes using matrix assisted laser desorption/ionization-time of flight-mass spectrometry (MALDI-TOF-MS). Electrophoresis. 1998; 19:1036–1045.
2. Yang HJ, Youn H, Seong KM, Jin YW, Kim J, Youn B. Phosphorylation of ribosomal protein S3 and antiapoptotic TRAF2 protein mediates radioresistance in non-small cell lung cancer cells. J Biol Chem. 2013; 288:2965–2975.
3. Kang J, Kim E, Kim W, Seong KM, Youn H, Kim JW, Kim J, Youn B. Rhamnetin and cirsiliol induce radiosensitization and inhibition of epithelial-mesenchymal transition (EMT) by miR-34a-mediated suppression of Notch-1 expression in non-small cell lung cancer cell lines. J Biol Chem. 2013; 288:27343–27357.
4. Son B, Jun SY, Seo H, Youn H, Yang HJ, Kim W, Kim HK, Kang C, Youn B. Inhibitory effect of traditional oriental medicine-derived monoamine oxidase B inhibitor on radioresistance of non-small cell lung cancer. Sci Rep. 2016; 6:21986.
5. Kim W, Kim E, Yang HJ, Kwon T, Han S, Lee S, Youn H, Jung Y, Kang C, Youn B. Inhibition of hedgehog signalling attenuates UVB-induced skin photoageing. Exp Dermatol. 2015; 24:611–617.
6. Livak KJ, Schmittgen TD. Analysis of relative gene expression data using real-time quantitative PCR and the 2(-Delta Delta C(T)) Method. Methods. 2001; 25:402–408.

Supplementary Table S1: Primers for determining expression levels of EMT-related genes

Gene name	Forward primer	Reverse primer
CDH1	5′- GGA TTG CAA ATT CCT GCC ATT C -3′	5′- AAC GTT GTC CCG GGT GTC A -3′
FN1	5′- TGA CCT TTT CTG GCT CGT CT -3′	5′- GTT CAG CAC AAA GGG CTC TC -3′
VIM	5′- GAC AAT GCG TCT CTG GCA CGT CTT -3′	5′- TCC TCC GCC TCC TGC AGG TTC TT -3′
GAPDH	5′- ATG ACA TCA AGA AGG TGG TG -3′	5′- CAT ACC AGG AAA TGA GCT TG -3′
